# Characteristics of Cadmium Sorption by Heat-Activated Red Mud in Aqueous Solution

**DOI:** 10.1038/s41598-018-31967-5

**Published:** 2018-09-10

**Authors:** Tianxue Yang, Lianxi Sheng, Yongfeng Wang, Kristen N. Wyckoff, Chunguang He, Qiang He

**Affiliations:** 1 State Environmental Protection Key Laboratory of Wetland Ecology and Vegetation Restoration, School of Environment, Northeast Normal University, Changchun, Jilin, China; 2 Department of Civil and Environmental Engineering, University of Tennessee, Knoxville, Tennessee USA; 3 Institute for a Secure and Sustainable Environment, University of Tennessee, Knoxville, Tennessee USA

## Abstract

Red mud as a waste material is produced in large quantities by the aluminum industry. Heat activation has been used to enhance sorption capacity of red mud for its beneficial reuse as an effective sorbent. In this study, heat-activated red mud (HARM) was investigated for its Cd(II) sorption capacity under various process conditions (Cd concentration, pH and contact time) using response surface methodology (RSM). Analysis with RSM identified pH as the most important process parameter. The positive correlation between higher pH and greater Cd(II) sorption was likely due to: (i) decreased proton competition with Cd(II) for sorption sites at higher pH; (ii) enhanced sorption via ion exchange by monovalent Cd species from hydrolysis at higher pH; and (iii) improved thermodynamics of sorption at higher pH as protons are being released as products. Further analysis indicated the sorption process was thermodynamically favorable with a negative change in Gibbs free energy. Additionally, the sorption process exhibited a positive change in enthalpy, indicative of endothermic nature of sorption; this is consistent with sorption increase at higher temperature. These findings provide needed insight into the mechanisms underlying Cd(II) sorption by HARM for more effective applications of heat-activated red mud as sorbents for Cd(II) removal.

## Introduction

Red mud is an abundant solid waste generated during the caustic digestion of bauxite ores with sodium hydroxide at increasing temperature and pressure for the production of alumina^1^. Red mud has shown characteristics as a promising sorbent due to its high surface reactivity^2^ and heterogeneous mineral composition^1,3,4^. Many studies have confirmed the sorption capacity of red mud for some anions such as phosphate^5,6^, fluoride^7^, and boron^8^. More importantly, red mud has been shown to be potentially effective in the sorptive removal of many cationic contaminants, particularly for certain toxic heavy metals^9–11^.

Cadmium (Cd) has been exerting a great pressure on the environment due to its increasingly higher input flux from anthropogenic sources like battery, alloy, and metal plating industries^12,13^. Moreover, Cd(II) is highly toxic to human. Intake of Cd(II) through drinking water damages the vital body organs like lungs^14^ and kidneys, which are two of the primary sites of injury after chronic Cd(II) exposure^15^. Cd(II) intake may further increase the risk of death from cancer, cardiovascular disease, and Alzheimer’s disease^15^; therefore it is necessary to develop effective remediation methods for Cd(II) contamination^12^. Sorption has proven to be an effective and economical method for the removal of Cd(II) in the aqueous environment^12,16^; thus using mineral wastes, red mud, as sorbents to remediate toxic Cd(II) should be a promising strategy.

Given the potential of red mud in the sorptive removal of heavy metals, efforts have been made to improving the sorption capacity of red mud. Heat activation has been proven to be an effective strategy to enhance the sorption capacity^1,17^. The effectiveness of heat activation in enhancing sorption performance has been conducted on a number of other heavy metals such as nickel^18^ and manganese^19^. Based on these studies, process parameters including pH, contact time, and adsorbate concentration have been shown to have a major influence on the sorption of heavy metals by heat-activated red mud. Due to the considerable differences in the investigated sorbates, as well as in the characteristics of starting red mud samples, the effects of process parameters on sorption were different; so it is necessary to study the Cd(II) sorption by heat-activated red mud which has seldom been reported.

The objectives of this work are to characterize Cd(II) sorption by heat-activated red mud using a mathematical model developed by response surface methodology (RSM) and to predict sorption behavior under various process conditions.

## Materials and Methods

### Preparation and characterization of heat-activated red mud

The original red mud samples were taken from the red mud disposal site of an alumina refinery in China. Samples were air-dried before passing through a 140-mesh sieve. Then 10 g portions were heated in an electrical furnace (Thermolyne, U.S.) at 200 °C, 400 °C, 500 °C, 800 °C and 900 °C for 3 hours, respectively. Since the result of Cd(II) sorption tests showed that the most effective red mud sample was the one obtained by heating at 500 °C (detailed results were provided in Results and Discussion section), denoted as HARM, samples heated to this temperature were selected for next analyses.

The mineralogical composition of HARM was studied by X-ray diffraction (XRD) analysis using a D2 PHASER Diffractometer with CuK*α* radiation, and a step/time scan mode of 0.75°/1 min. The crystalline phase was identified by comparing the XRD patterns with standards available in the powder diffraction file (PDF2) database. XPS data were acquired with a Phoibos 150 with Al Ka radiation (SPECS, U.S.). The binding energy correction due to the charging effects has been based on the main contribution of the carbon C1s line, assuming it corresponds to adventitious carbon at 284.8 eV. Scanning electron microscopy (SEM) was performed using a Zeiss LEO 1525 SEM. Particle size analysis of the components of HARM was obtained using the laser diffraction method with a Malvern Mastersizer S instrument, long bench with 300RF lens. The specific surface area of HARM was determined by Brunauer–Emmett–Teller (BET)/N_2_ adsorption method using an automatic specific surface area measurement (Belsorp-max, MicrotracBEL, Japan). To determine the pH of HARM, 1 g of air-dried HARM was mixed with 5 mL of deionized water for 5 minutes and left to stand for 30 minutes. The supernatant was used to measure pH with a combination pH electrode (Oakton pH 700, U.S.). The batch equilibrium method was used for the determination of the point of zero charge (pH_PZC_) of HARM^20^. 0.1 g of dried HARM was shaken at 250 rpm in a glass vial for 24 h with 20 mL of 0.1 mol∙L^−1^ NaCl solution of a known initial pH. Initial pH values of solution were adjusted in a wide pH range (from 1 to 11) by adding either 0.1 mol∙L^−1^ HCl or 0.1 mol∙L^−1^ NaOH. The pH_PZC_ values were determined using the plateau of a graph of the final pH against the initial pH.

### Sorption experiments

Sorption was tested in batch conditions by shaking suspensions of 0.01 g of red mud and 20 mL of CdCl_2_ solution in closed glass bottles on a horizontal laboratory shaker (New Brunswick Scientific Co., Inc, U.S.) at a constant speed of 250 rpm. Each process parametric sorption experiment was tested in triplicate. After each set of sorption experiments, the liquid phase was separated from the solid residue by centrifugation (SORVALL RC 6, Thermo Scientific, U.S.) for 15 min at 14000 rpm, and the remaining Cd(II) concentrations (C_e_) were determined by ICP-OES (iCAP 7000, ThermoFisher Scientific, U.S.). Sorption efficiency was evaluated by calculating the amount of metal sorbed (*q*_*e*_, mg∙g^−1^) (Equation 1).

1 $${q}_{e}=({C}_{0}-{C}_{e})\times \frac{V}{m}$$

where *V* (L) is solution volume, *m* (g) is red mud sample mass, *C*_0_ (mg∙L^−1^) is initial Cd(II) concentration, and *C*_*e*_ (mg∙L^−1^) is equilibrium Cd(II) concentration in the solution.

#### Effect of heat treatment

The maximum Cd(II) sorption capacities of original red mud and red mud heat-treated at different temperatures (200–900 °C) were evaluated through batch sorption experiments. The sorbent dosage, initial pH, temperature, initial Cd(II) solution concentration, and contact time were 0.5 g∙L^−1^, 6, 20 °C, 200 mg∙L^−1^ and 24 hours, respectively.

#### Effect of pH

The effect of initial pH was examined using HARM at 20 °C and a contact time of 24 h. The initial Cd(II) concentration was 10 mg∙L^−1^, and initial pH of the suspension was adjusted between 2–8 at the beginning of the experiment by adding a negligible volume of 1 mol∙L^−1^ or 0.1 mol∙L^−1^ HCl. After sorption, the solid phase was separated from the solution by centrifugation, and final pH value was measured in the supernatant.

#### Effect of Cd(II) concentration and reaction temperature

The impacts from the Cd(II) concentration and the reaction temperature were evaluated at an initial pH 6, and contact time of 24 h. Batch sorption experiments were conducted at 20 °C, 30 °C and 40 °C with varying initial Cd(II) concentrations ranging from 1 mg∙L^−1^ to 227 mg∙L^−1^. The final pH was measured as the last section.

### Response surface methodology modeling

Response surface methodology (RSM) is a mathematical model used to predict sorption behavior and to evaluate the relative importance and interaction of each parameter^21^. In this study, the 3-factor 3-level Box-Behnken experimental design (BBD) was applied in the RSM model. The BBD design was capable of streamlining experimental setup^22^. Table 1 shows the results of sorption experiments which were conducted on the base of BBD. The analysis of variables was evaluated by Design-Expert V10.0 (Stat-Ease Inc., U.S.). The quadratic equation model was used in RSM modeling (Equation 2).

2 $${Y}={\beta }_{0}+\sum _{{\rm{i}}={\rm{1}}}^{k}{\beta }_{i}{x}_{i}+\sum _{{i}=1}^{{k}}{\beta }_{{\rm{ii}}}{x}_{{\rm{ii}}}^{2}+\sum _{{i}=1}^{{k}}\sum _{{j}=1}^{{k}}{\beta }_{{\rm{ij}}}{x}_{i}{x}_{j}$$

where *Y* is the process response, *k* is the number of the patterns, *i* and *j* are the index numbers for pattern, *β*_0_ is the offset term, *β*_i_ is the linear effect of the input factor *x*_i_, *β*_ii_ is the quadratic effect of input factor *x*_i_, and *β*_ij_ is the interaction effect^23^.

**Table 1:** Box-Behnken experimental design for RSM model.

Level	Initial Cd(II) concentration (mg∙L^−1^)	Initial pH	Time (h)
Low level (−1)	1	2	1
Center level (0)	102	4	12.5
High level (1)	203	6	24

## Results and Discussion

### Effect of heat treatment on red mud Cd(II) sorption capacity

**Figure 1:**
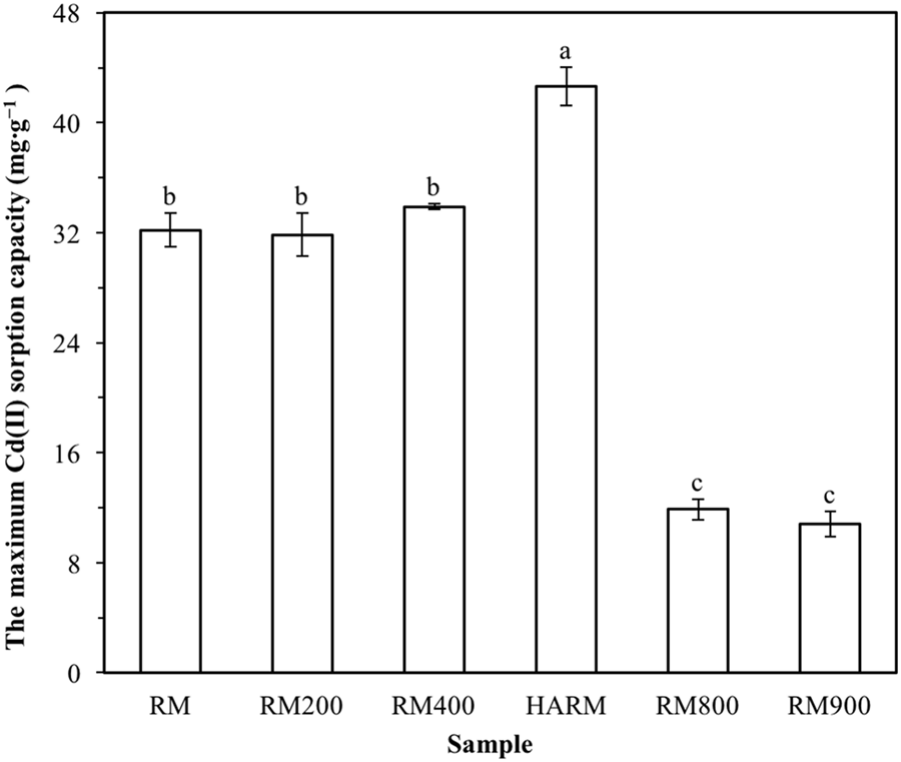
The maximum Cd(II) sorption capacities of original red mud (RM), red mud heat-activated at 500 °C (HARM) and red mud heat-treated at 200, 400, 800, 900 °C (RM200–RM900). The means are not significantly different from each other in columns labeled with the same lowercase letters (ANOVA, LSD test, *p* < 0.05).

The maximum sorption capacities of all red mud samples in this study were evaluated through batch sorption experiments. The sorbent dosage, initial pH and temperature were 0.5 g∙L^−1^, 6 and 20 °C, respectively. In order to reach maximum sorption, the initial Cd(II) solution concentration and contact time were fixed at the high level (200 mg∙L^−1^ and 24 hours, respectively). According to Fig. 1, compared with other red mud samples in this work, red mud heat-activated at 500 °C (HARM) exhibited the highest sorption capacity. From Table 2, compared with other industrial wastes tested under room temperature (20–25 °C) in previous literature, HARM showed higher Cd(II) sorption capacity, although the sorption capacity of HARM was lower than the Cd(II) sorption capacities of some synthetic materials or/and the modified typical sorbents such as activated carbon and metal oxides^24^. So, HARM was selected for further investigations in this study.

**Table 2:** A comparison of maximum sorption capacities (*q*_*max*_) (mg∙g^−1^) for Cd(II) ions by various sorbents under room temperature (20–25 °C).

Sorbent	*q*_*max*_	Sorbent	*q*_*max*_
Coal fly ash^41^	0.089	β-cyclodextrin polymers^42^	136.43
Lignin^43^	25.40	Multi-functional cotton fiber^44^	182.27
Kraft lignin^45^	8.21	Magnetic iron oxide nanoparticles loaded sawdust carbon^46^	51
NaOH-treated fly ash^47^	30.21	EDTA modified Fe_3_O_4_/sawdust carbon nanocomposites^46^	63.3
Iron oxide activated red mud^14^	0.116	Alumina nanoparticles^48^	1.86
TiO_2_/fly ash^49^	35.80	Glycerol-modified alumina^48^	0.67
Acidified red mud^50^	12.07	Synthetic mineral^51^	47
Balling milling nano-particle red mud^9^	23.61	Amino-functionalized activated carbon^52^	79.2
Acidified red mud^9^	21.36	Thiol-functionalized activated carbon^52^	130.05
Original red mud^9^	17.99	Magnesium silicate-hydrothermal carbon^53^	108
Heat-activated red mud (This work)	42.74		

### Characteristics of heat-activated red mud

**Figure 2:**
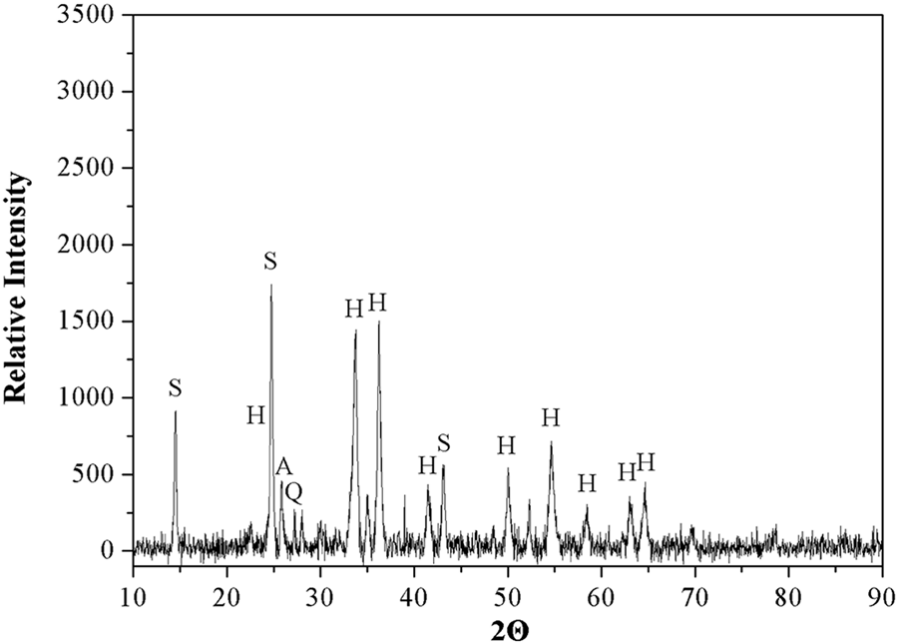
XRD pattern of heat-activated red mud (A-anatase, H-hematite, Q-quartz, S-sodalite).

**Figure 3:**
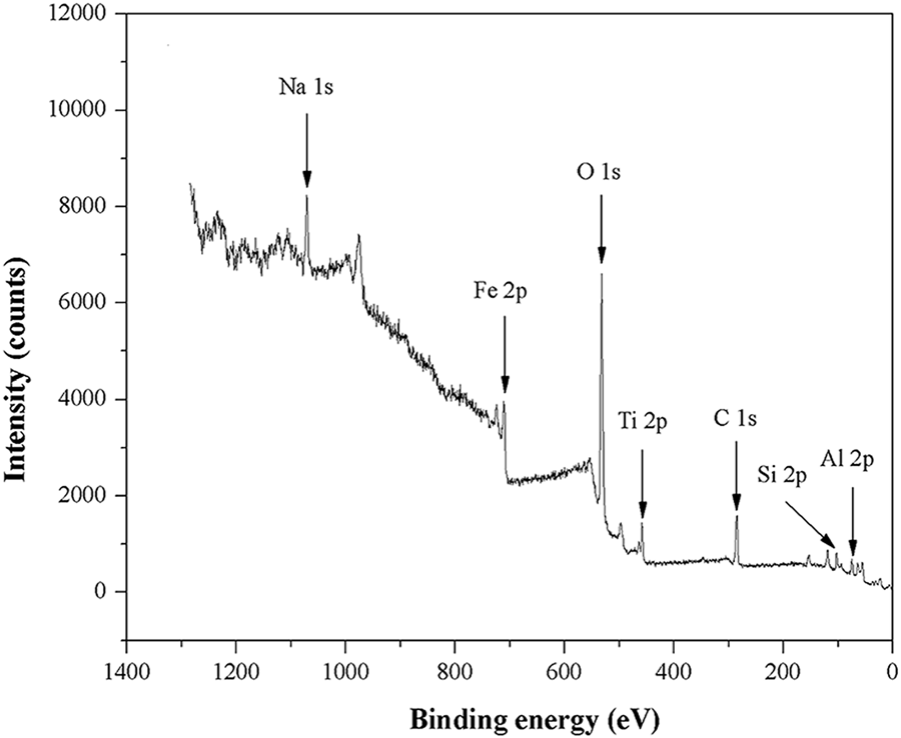
XPS spectrum of heat-activated red mud.

**Figure 4:**
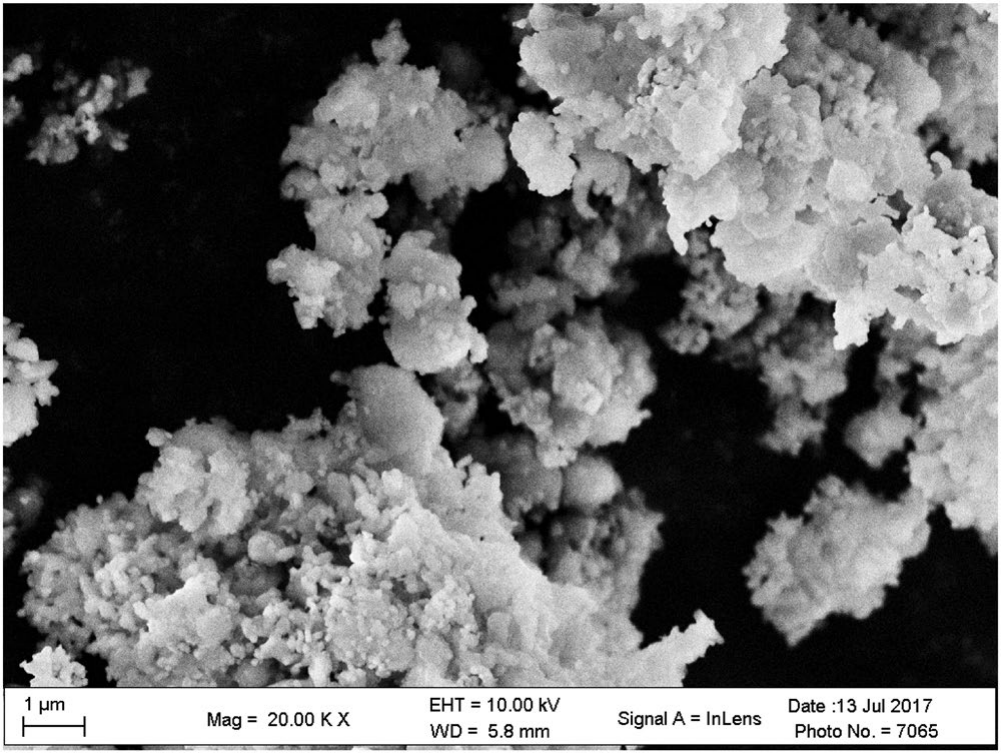
SEM image of heat-activated red mud.

The mineralogical composition of HARM was investigated using XRD analysis (Fig. 2). The main crystalline phases were determined to be hematite (Fe_2_O_3_) and sodalite (Na_8_Si_6_Al_6_O_24_Cl_2_), which was consistent with findings from previous studies on the XRD patterns of heat-treated red mud samples^18,25^. Additional peaks detected in the XRD diffractogram also indicated the presence of anatase (TiO_2_) and quartz (SiO_2_) phases in the HARM. As shown in XPS spectrum of HARM (Fig. 3), Fe, Al, O, Na, C, Ti and Si were detected on the surface of RM500, which was consistent with XRD analyzed above (Fig. 2). The morphologies of HARM particles were investigated by scanning electron microscopy (SEM) shown in the Fig. 4. HARM contained various particles with different size and shape (Fig. 4). Particle size analysis of HARM showed that over 90% of particles had a diameter smaller than 50 μm (Table 3). Based on soil texture classification^26^, this HARM could be classified as a silty clay. Table 3 shows the specific surface area of 32.77 m^2^∙g^−1^, and mean pore diameter and total pore volume were 5.37 nm and 44 mm^3^∙g^−1^, respectively. The HARM samples tested in this study exhibited high alkalinity with a pH of approximately 10.9. The pH_PZC_ value for HARM was approximately 10.6 which was determined from the plateau region of the initial pH vs. final pH plot (Supplementary Fig. S1).

**Table 3:** Particle size analysis, specific surface area (BET), mean pore diameter, and total pore volume of heat-activated red mud.

Diameter Volume (%)			Specific surface area (m^2^∙g^−1^)	Mean pore diameter (nm)	Total pore volume (mm^3^∙g^−1^)
<2 μm	2 μm–50 μm	>50 μm	32.77	5.37	44
51.29	44.78	3.92			

### Modeling by heat-activated red mud with response surface methodology (RSM)

#### Model development with RSM and statistical evaluation

The sorption of Cd(II) was expected to be impacted by the Cd(II) concentration, pH, and contact time for the sorption process. It is expected that Cd(II) concentration and pH will deviate with sorption process. For modeling, initial Cd(II) concentration and initial pH were used. Therefore, initial Cd(II) concentration and initial pH would be referred to as Cd(II) concentration and pH, respectively. A regression model was developed to investigate the effect of these three parameters on the Cd(II) sorption behavior to heat-activated red mud using the response surface methodology (RSM). A Box-Behnken experimental design (BBD) was used to develop the experimental matrix of 15 sorption experiments with Cd(II) concentration, pH, and contact time as the independent variables (Table 4). Application of RSM yielded the following equation to describe sorption of Cd(II) to heat-activated red mud (Equation 3):

3 $$\begin{array}{ccc}{\rm{Y}} & = & 28.16-0.031{{\rm{X}}}_{1}-13.35{{\rm{X}}}_{2}-1.38{{\rm{X}}}_{3}+0.032{{\rm{X}}}_{1}\cdot {{\rm{X}}}_{2}\\  &  & +4.2\times {10}^{-3}{{\rm{X}}}_{1}\cdot {{\rm{X}}}_{3}+0.12{{\rm{X}}}_{2}\cdot {{\rm{X}}}_{3}\\  &  & -3.75\times {10}^{-4}{{\rm{X}}}_{1}^{2}+\,1.65{{\rm{X}}}_{2}^{2}\\  &  & +0.034{{\rm{X}}}_{3}^{2}\end{array}$$

where Y is the predicted amount of Cd(II) sorbed (mg∙g^−1^); X_1_, X_2_, and X_3_ represent the three independent variables — Cd concentration (mg∙L^−1^), pH, and contact time (h), respectively.

**Table 4:** Box-Behnken design matrix with three independent variables expressed in coded and natural units. *q*_e_: The amount of Cd(II) sorbed.

Run	Cd concentration (mg∙L^−1^)		pH		Time (h)		*q*_*e*_ (mg∙g^−1^)
	Coded	Uncoded	Coded	Uncoded	Coded	Uncoded	Measured
1	−1	1	−1	2	0	12.5	0.33 ± 0.00
2	−1	1	1	6	0	12.5	2.15 ± 0.00
3	1	203	−1	2	0	12.5	3.49 ± 0.80
4	1	203	1	6	0	12.5	31.55 ± 1.02
5	−1	1	0	4	−1	1	0.31 ± 0.01
6	−1	1	0	4	1	24	0.34 ± 0.01
7	1	203	0	4	−1	1	4.39 ± 0.10
8	1	203	0	4	1	24	23.93 ± 0.45
9	0	102	−1	2	−1	1	5.72 ± 0.58
10	0	102	−1	2	1	24	7.34 ± 0.82
11	0	102	1	6	−1	1	22.46 ± 0.54
12	0	102	1	6	1	24	35.13 ± 1.47
13	0	102	0	4	0	12.5	6.36 ± 0.69
14	0	102	0	4	0	12.5	5.92 ± 0.02
15	0	102	0	4	0	12.5	7.55 ± 0.32

The analysis of variance (ANOVA) was conducted to test the significance of the fit of the established quadratic model for the experimental data, and significant effect of the terms in the model on the response (Table 5). ANOVA is a statistical technique that subdivides the total variation in a set of data into component parts associated with specific sources of variation for the purpose of testing hypotheses on the parameters of the model^27^. The ANOVA of the regression model (Equation 3) indicated that the model could explain a considerable amount of the variation in the dependent variable (the amount of Cd(II) sorbed) with 95% certainty, as was evident from the high *F* value (30.16) which was higher than the tabulated *F* value (*F*_0.05, 9, 5_ = 4.77) at the 5% level (Table 5). Further, the *p*-value (0.0008) was lower than 0.05, indicating that this quadratic model was statistically significant^27,28^. The coefficient of determination (*R*^2^) (Equation 4) describes the amount of variation in the observed responses that can be explained by the model^29^. In this case, *R*^2^ was 98.19% indicating that only 1.81% of total variations could not be explained by this model.

4 $${R}^{2}={{\rm{SS}}}_{{\rm{model}}}/({{\rm{SS}}}_{{\rm{model}}}+{{\rm{SS}}}_{{\rm{residual}}})\,$$

where SS is sum of squares.

**Table 5:** ANOVA of the RSM model. X_1_, X_2_, and X_3_ are the actual terms for three independent test variables — Cd(II) concentration, pH, and contact time, respectively.

Factors	Statistics					
	Sum of squares	Degrees of freedom	Mean square	*F* value	*p*-value prob > *F*	Remark
Model	1884.73	9	209.41	30.16	0.0008	significant
X_1_	453.54	1	453.54	65.32	0.0005	
X_2_	692.18	1	692.18	99.69	0.0002	
X_3_	143.16	1	143.16	20.62	0.0062	
X_1_X_2_	172.08	1	172.08	24.78	0.0042	
X_1_X_3_	95.24	1	95.24	13.72	0.0139	
X_2_X_3_	30.53	1	30.53	4.40	0.0901	
X_1_^2^	53.99	1	53.99	7.78	0.0385	
X_2_^2^	160.60	1	160.60	23.13	0.0048	
X_3_^2^	73.36	1	73.36	10.57	0.0227	
Residual	34.72	5	6.94			
Lack of fit	33.31	3	11.10	15.74	0.0603	not significant
Pure error	1.41	2	0.71			
Cor. total	1919.44	14				

#### Validation of Cd(II) sorption model

**Figure 5:**
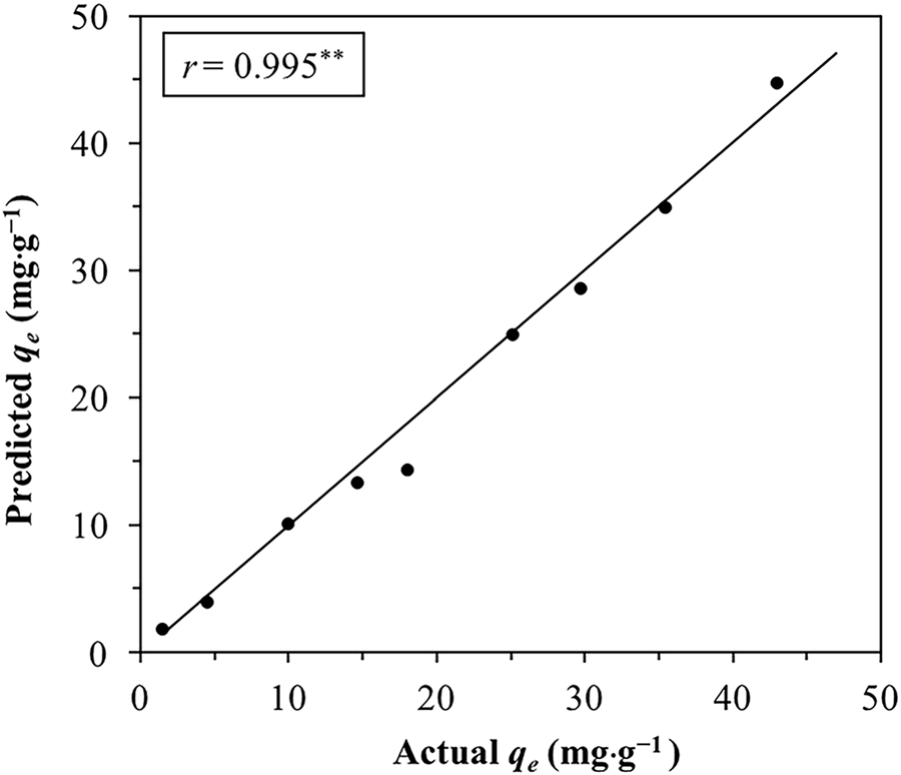
Correlation between Cd(II) sorption experimental result and model prediction. The diagonal line indicates equality between experimental result and model prediction. *q*_*e*_ is the amount of Cd(II) sorbed. *r* (correlation coefficient) is a parameter of Pearson’s correlation analysis between experimental result and model prediction. **Indicates correlation is significant at 0.01 level.

To further validate the Cd(II) sorption model (Equation 3) developed with RSM, additional sorption experiments were conducted to compare actual Cd(II) sorption performance with model prediction (Supplementary Table S1). The distributions of experimental results and model predictions were close to normal based on the Kolmogorov-Smirnov Test^30^; therefore a two-sample, unpaired *t*-test was conducted to test the difference between experimental results and those predicted by the sorption model (Equation 3). Since *p* > α (0.93 > 0.05), the experimental results and model predictions were not considered to be significantly different^22^, indicating the sorption model (Equation 3) provided a good fit to experiment results. The validity of the sorption model was also evident from the strong correlation between experimental result and model prediction according to Pearson’s correlation analysis (Fig. 5). Further, the model predictions and experimental results were very similar in value (Fig. 5), suggesting the accuracy of the sorption model in describing Cd(II) sorption behavior.

#### Effect of model components and their interactions on Cd(II) sorption

X_1_, X_2_, X_3_, X_1_X_2_, X_1_X_3_, X_1_^2^, X_2_^2^ and X_3_^2^ were significant model terms based on the *p*-value of each component (Table 5). The sum of square (SS) of each component obtained from ANOVA quantifies the importance of each component in the sorption process. As the value of the SS increases, the significance of the corresponding component in the undergoing process also increases^29^. Based on the SS obtained from the ANOVA (Table 5), the percent contribution of each RSM model component to Cd(II) sorption (PC) was calculated (Equation 5):

5 $${{\rm{PC}}}_{{\rm{i}}}={{\rm{SS}}}_{{\rm{i}}}/\sum _{j=1}^{c}{{\rm{SS}}}_{j}$$

where SS_i_ is the sum of squares of i component of the model, and c is the total number of components of the model.

**Figure 6:**
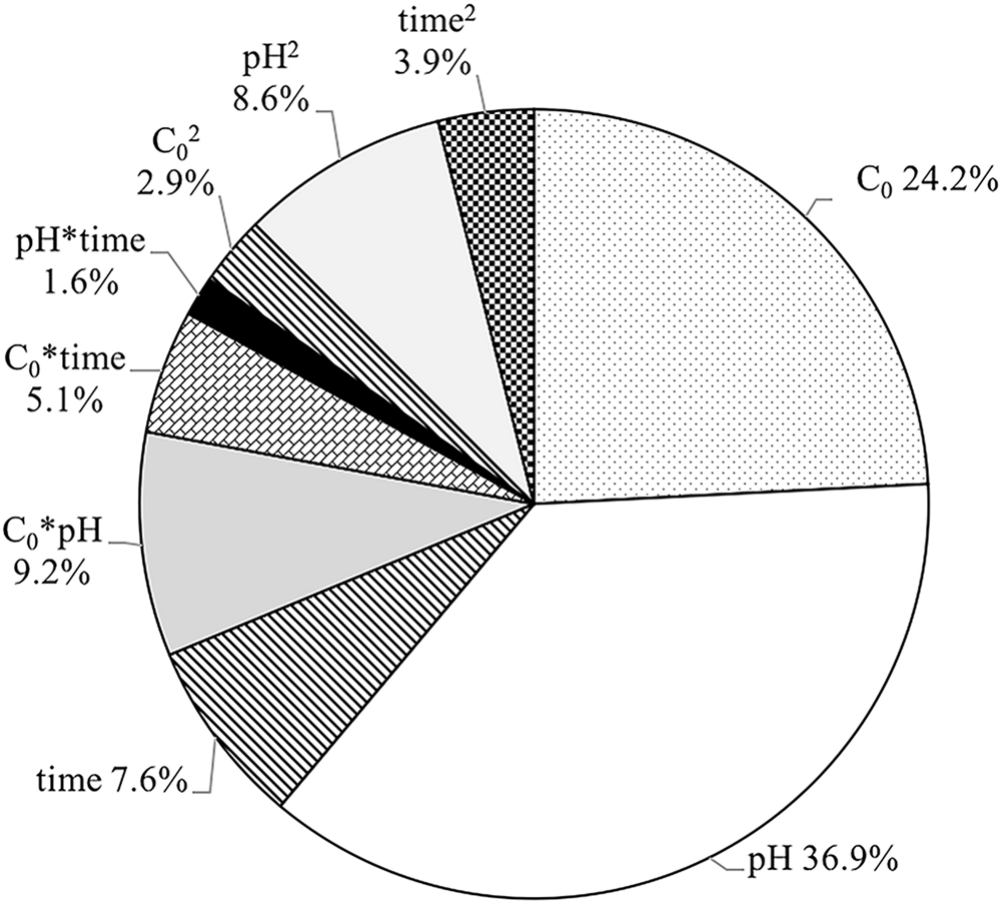
Percent contribution of RSM model components to Cd(II) sorption.

From examining the PC values of the components, the first-order term showed the highest level of significance, followed by interaction and quadratic terms (Fig. 6). pH was dominant with a contribution of 36.9%, followed by Cd(II) concentration and contact time (Fig. 6).

**Figure 7:**
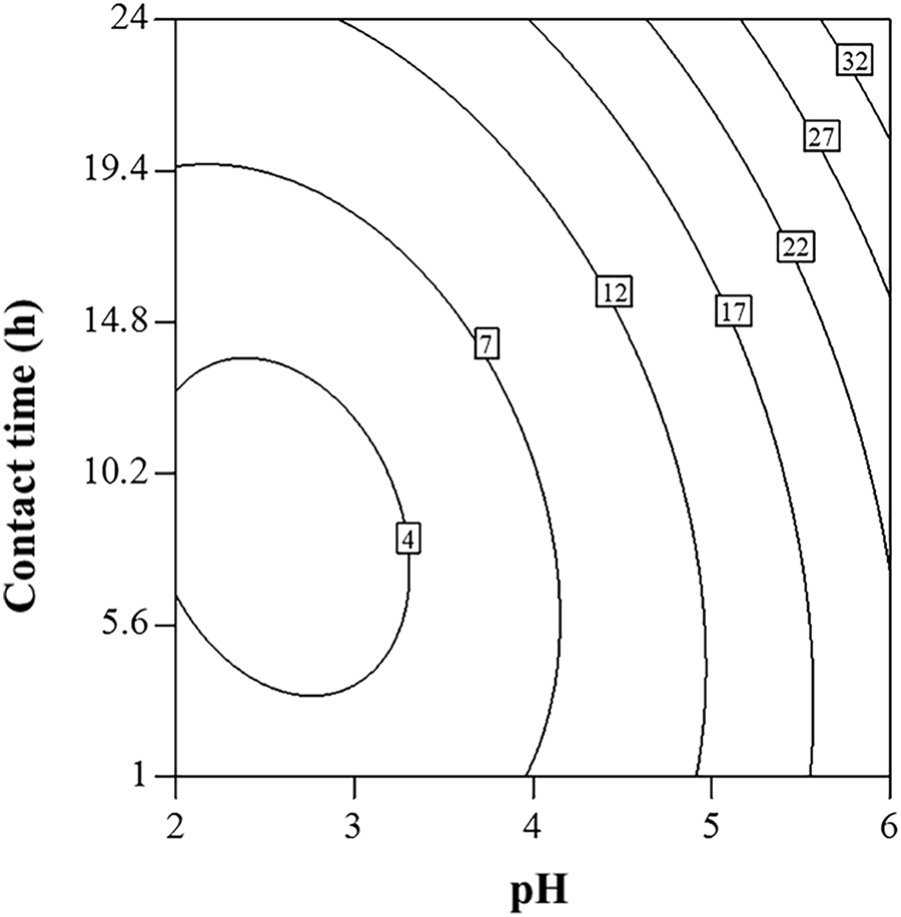
Contour plot showing the impact on Cd(II) sorption by the process variable pair of pH and contact time with Cd(II) concentration at 102 mg∙L^−1^. The numerical labels on the contour lines indicate the amount of Cd(II) sorption (mg∙g^−1^).

To further explore the effects of pH on the Cd(II) sorption behavior, contour plot was carried out (Fig. 7). Contour plots of experimental factors can only show two factors at a time, the other factor which was not included in the figure was held at the center level (Cd(II) concentration = 102 mg∙L^−1^). In general, pH had a positive correlation with Cd(II) sorption. In the pH ranged from 2 to 4, Cd(II) sorption capacity was relatively low. However, the considerable increase in sorption was observed at higher pH. To elucidate the mechanism of the effect of pH on the sorption, other batch sorption experiments and chemical analysis were conducted.

### The effect of pH on Cd(II) sorption

**Figure 8:**
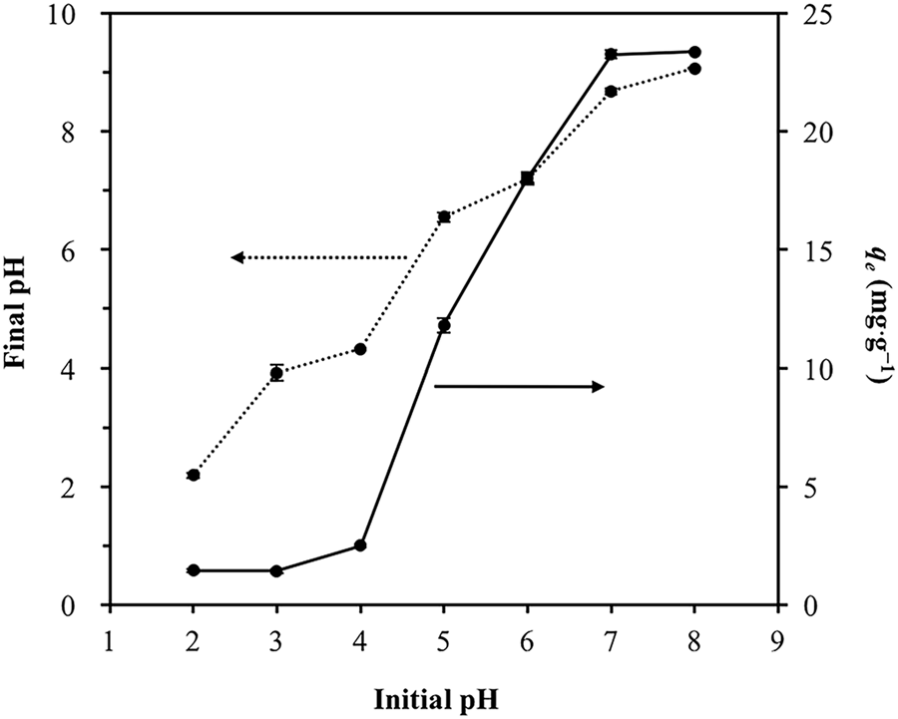
Effect of initial pH on the amount of Cd(II) sorbed (*q*_*e*_) (solid line) and final pH (dashed line). Error bars indicate the standard deviations.

The batch sorption experiments were conducted to further confirm and try to explain the effect of pH on the Cd(II) sorption by HARM. Initial Cd(II) concentration, contact time, HARM dosage and reaction temperature were 10 mg∙L^−1^, 24 h, 0.5 g∙L^−1^ and 20 °C, respectively. The initial pH was adjusted between 2–8. From Fig. 8, the amount of Cd(II) sorbed onto HARM (*q*_*e*_) increased with an increase of pH was consistent with the RSM result. This is in agreement with previous research on the effect of pH on the sorption of metal cations to red mud^14,18,19^ and other adsorbents^31,32^. At lower pH, the amount of Cd(II) sorbed was relatively low due to competitive sorption between more H^+^ and Cd(II) cations (Fig. 8). However, at higher pH, with less H^+^ in the solution, the competitive sorption was weak, contributing to a higher sorption efficiency.

**Figure 9:**
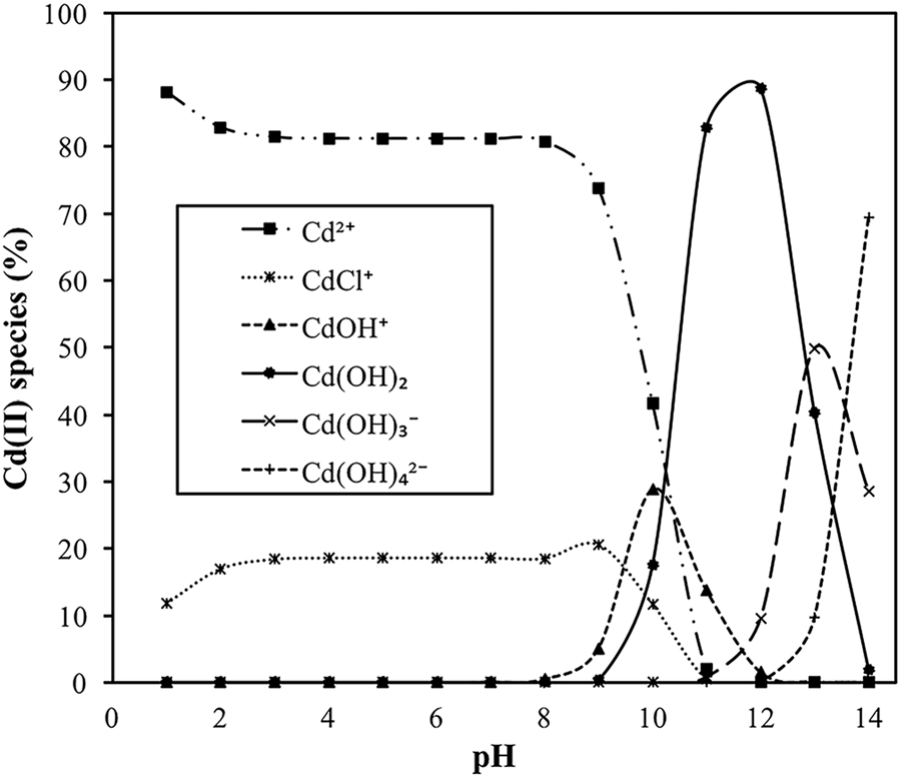
Distribution of various Cd(II) species as a function of pH.

Moreover, the distribution of the Cd(II) speciation in the solution which is controlled by pH also has an effect on Cd(II) sorption onto HARM. Using appropriate Cd^2+^ and Cl^−^ concentrations, Visual MINTEQ software (EPA, U.S.) was applied for the calculation of the speciation of Cd(II) vs. pH (Fig. 9). At higher pH (pH > 8), the hydrolysis of Cd^2+^ initiates contributing to the increase in the percentage of monovalent Cd species (CdCl^+^ and CdOH^+^) (Fig. 9). As a result, sorption efficiency was enhanced as greater Cd(II) sorption occurred via ion exchange, when equilibrium pH (final pH) exceeded 8 (Fig. 8). For further verification of this result, the effect of pH on ion exchange process during Cd(II) sorption, and the mechanism of ion exchange were explored based on the number of exchangeable metal cations released from HARM when the Cd(II) was sorbed onto HARM with different pH. Two sorption experiments were conducted at 24 h (contact time), 0.5 g∙L^−1^ (dose), 20 °C (reaction temperature), and 200 mg∙L^−1^ (initial Cd(II) concentration). The initial pH values of these two sorption experiments were 6 and 8, respectively where the corresponding final pH values were 7 and 9. In order to suppress the effect of pH on HARM dissolving, two control experiments were carried out in the absence of Cd(II) with the same experimental procedure and the same final pH (7 and 9, respectively) as the sorption experiments. Distilled water (DI water) was used as reference instead of Cd(II) solution. After equilibration, the mixtures HARM/DI water and HARM/Cd(II) solution were centrifuged, and the concentrations of cations including Na^+^, Ca^2+^, K^+^, Mg^2+^ and Cd(II) in the supernatant were recorded (Table 6). The results showed K^+^ and Mg^2+^ were not detected. Compared with control, it was observed that Cd(II) sorption was accompanied by the stoichiometric release of the Na^+^ and Ca^2+^ (Table 6). The concentrations of Na^+^ and Ca^2+^ released on the Cd(II) uptake by HARM were used to calculate the number of positive charges released through ion exchange (Equation 6).

6 $$\begin{array}{l}{\rm{The}}\,{\rm{number}}\,{\rm{of}}\,{\rm{positive}}\,{\rm{charges}}\,{\rm{released}}\\ \begin{array}{rcl} & = & {\rm{Cd}}({\rm{I}}{\rm{I}})\,sorption({{\rm{Na}}}_{{\rm{released}}}^{+}+2{{\rm{Ca}}}_{{\rm{released}}}^{2+})\\  &  & -\,{\rm{Control}}({{\rm{Na}}}_{{\rm{released}}}^{+}+2{{\rm{Ca}}}_{{\rm{released}}}^{2+})\end{array}\end{array}$$

**Table 6:** The concentrations of metal cations released from heat-activated red mud (HARM) on the Cd(II) uptake by HARM under different conditions. pH: final pH.

pH	Cd(II) sorption capacity (mg∙g^−1^)	Concentrations of released exchangeable metal cations (mmol∙g^−1^)		
7	42.64	Experiment	Na^+^	Ca^2+^
		Control	1.591	0.061
		Cd(II) sorption	1.610	0.065
9	55.19	Experiment	Na^+^	Ca^2+^
		Control	1.025	0
		Cd(II) sorption	1.054	0

Control experiment conditions: sorbent dose = 0.5 g∙L^−1^, initial Cd(II) concentration = 0 (distilled water), and temperature = 20 °C, contact time = 24 hours. Cd(II) sorption conditions: sorbent dose = 0.5 g∙L^−1^, initial Cd(II) concentration = 200 mg∙L^−1^, and temperature = 20 °C, contact time = 24 hours.

The number of positive charges released at final pH 7 and it at final pH 9 were similar (0.027 and 0.029 mmol∙g^−1^, respectively). However, at pH 7, Cd^2+^ cations are prevalent ( > 80%), and at pH 9, the fraction of Cd^2+^ cations decreased, and the percentage of monovalent Cd species (CdCl^+^ and CdOH^+^) increased (Fig. 9) contributing to the stronger ion exchange. So, it could be concluded that the ion exchange process was stronger at higher pH due to the increase in the percentage of monovalent Cd species which contributed to the higher sorption capacity shown in Table 6. From Fig. 9, the precipitation of Cd(OH)_2_ could contribute to the Cd ion removal, where equilibrium pH (final pH) was greater than 9.

**Figure 10:**
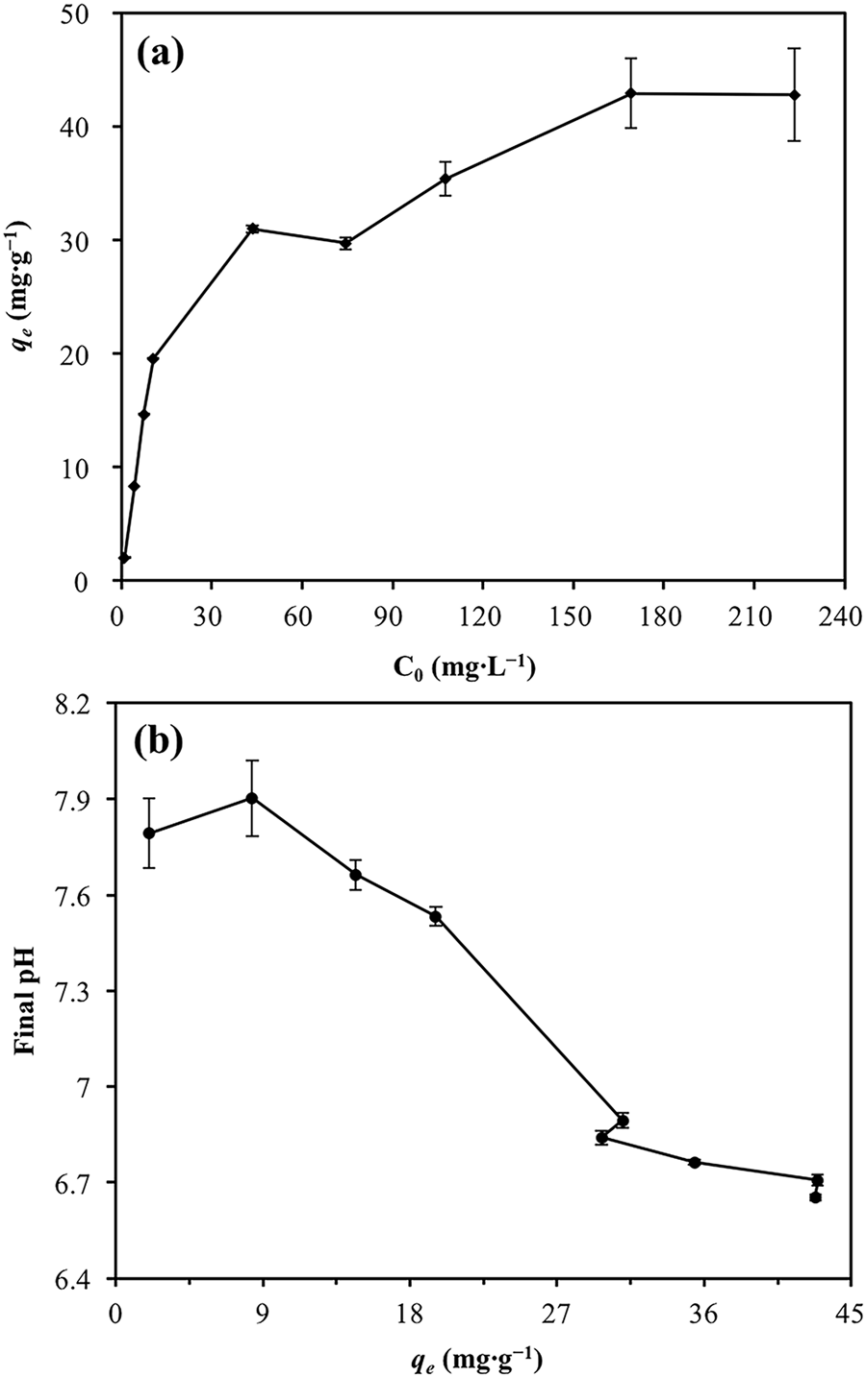
(**a**) The relationship between initial Cd(II) concentration (C_0_) and the amount of Cd(II) sorbed (*q*_*e*_). (**b**) The relationship between the amount of Cd(II) sorbed (*q*_*e*_) and final pH. Error bars indicate standard deviations.

The effect of pH on the Cd(II) sorption can be interpreted through other batch sorption experiments (1.15 mg∙L^−1^–227.34 mg∙L^−1^ initial Cd(II) concentration, 0.5 g∙L^−1^ HARM dosage, 24 h contact time, 6 initial pH and 20 °C reaction temperature). The results indicated that Cd(II) sorption onto HARM (*q*_*e*_) increased with initial Cd(II) concentration increase, then reached saturation sorption capacity (Fig. 10a). The increased sorption capacity of Cd(II) was followed by considerable proton release, resulting in a decrease in final pH (Fig. 10b). This demonstrated that based on sorption capacity increase, more H^+^ on the surface of HARM was replaced by Cd^2+^ through specific cation sorption^18^. Specific sorption can be expressed by surface complexation model demonstrating that Cd^2+^ could be sorbed to the hydroxyl functional groups of red mud and the H^+^ could be replaced by Cd ions^33–35^ (Eqs 7 and 8).

7 $${{\rm{Cd}}}^{2+}+{\rm{SOH}}+{{\rm{H}}}_{2}{\rm{O}}\rightleftharpoons {\rm{SOCdOH}}+2{{\rm{H}}}^{+}$$

and/or

8 $${{\rm{Cd}}}^{2+}+{\rm{SOH}}\rightleftharpoons {{\rm{SOCd}}}^{+}+{{\rm{H}}}^{+}$$

where SOH represents the functional groups of Fe– and Al–oxyhydroxide mineral phases in red mud.

Equations 7 and 8 indicated another reason for the positive relationship between higher pH and greater sorption capacity (Fig. 8). At lower pH, more H^+^ in the solution suppressed the specific sorption process through increasing in *ΔG* of the specific sorption.

### The effect of reaction temperature on Cd(II) sorption

In order to elaborate the feasibility of the sorption process, thermodynamic studies were carried out based on the effect of reaction temperature on the Cd(II) sorption at different initial Cd(II) concentration levels. Moreover, isotherm study at different reaction temperature could confirm the feasibility of the sorption, while also provide a mechanistic understanding of sorption.

**Figure 11:**
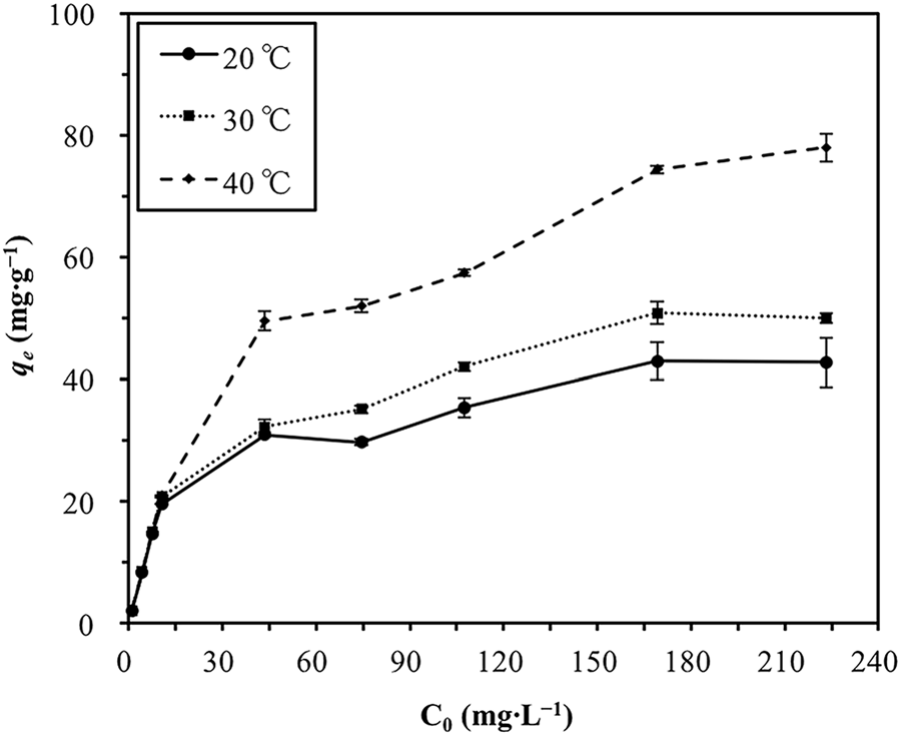
Effect of initial Cd(II) concentration (C_0_) and reaction temperature on the amount of Cd(II) sorbed (*q*_*e*_). Error bars indicate standard deviations.

In isotherm and thermodynamic studies, different initial Cd(II) concentrations (1.15 mg∙L^−1^–227.34 mg∙L^−1^) were used in conjunction with a 24 h contact time, an initial pH of 6, a HARM dose of 0.5 g∙L^−1^, and three different temperatures (20 °C, 30 °C and 40 °C) to perform the sorption experiments. At higher initial Cd(II) concentrations, increasing reaction temperature could enhance the sorption capacity (Fig. 11); however at lower initial concentrations (C_0_ < 11 mg∙L^−1^), the amount of Cd(II) sorbed (*q*_*e*_) was independent of reaction temperature. This may be because when compared with the lower Cd(II) sorbates concentration, sorption sites on the surface of HARM were sufficient causing the decrease of sorption enhancement by higher temperature.

The Langmuir isotherm model was used for equilibrium data fitting (Equation 9)^36^.

9 $$\frac{{C}_{e}}{{q}_{e}}=\frac{{C}_{e}}{{q}_{0}}+\frac{1}{{q}_{0}b}$$

where *q*_*e*_ (mg∙g^−1^) and *C*_*e*_ (mg∙L^−1^) denote the equilibrium concentrations of Cd(II) in the solid and liquid phases, respectively, *q*_0_ (mg∙g^−1^) is the maximum sorption capacity, *b* (L∙mg^−1^) is the Langmuir constant related to the sorption energy.

The dimensionless separation factor *R*_*L*_ shows the feasibility of the process (Equation 10)^37^.

10 $${R}_{L}=\frac{1}{1+{C}_{0}b}$$

The *R*_*L*_ values lie in the range of 0–1, demonstrating the sorption is a favorable process^14,38^.

The values of *q*_0_ and *b* in the Langmuir isotherm model were evaluated from the slope and intercept of plots of C_e_/*q*_*e*_ vs. C_e_ at 20 °C, 30 °C and 40 °C (Supplementary Fig. S2) (Table 7). The Langmuir model had a higher regression coefficient (*R*^2^), indicating that sorption corresponds to a monolayer formation of Cd(II) onto the HARM surface^14^ (Table 7). The maximum sorption capacity (*q*_0_) and the sorption capacity at unit concentration (*k*_*F*_) increased with increasing temperature, indicating the endothermic nature of sorption. The calculated *R*_*L*_ values were in the range of 0–1, indicating favorable sorption of Cd(II) onto HARM^39^.

**Table 7:** Parameters of Langmuir model for Cd(II) sorption onto heat-activated red mud. *q*_0_: the maximum sorption capacity calculated from Langmuir model. *b*: the Langmuir constant related to the sorption energy. *R*_*L*_: the feasibility of the process.

Temp. (°C)	*q*_0_ (mg∙g^−1^)	*b* (L∙mg^−1^)	*R*_*L*_ (range)	*R* ^2^
20	42.74	0.160	0.03–0.85	0.98
30	50.25	0.162	0.03–0.85	0.98
40	75.76	0.176	0.03–0.84	0.97

The change in free energy (*ΔG*^0^ (kJ∙mol^−1^)) was calculated using Langmuir constant, *b* (Equation 11). The values of change in enthalpy (*ΔH*^0^ (kJ∙mol^−1^)) and entropy (*ΔS*^0^ (kJ∙mol^−1^)) were calculated from the intercept and slope of the linear plot of *ΔG*^0^ vs. *T* (Equations 11 and 12) (Supplementary Fig. S3). Negative *ΔG*^0^ values indicated the spontaneous nature of the sorption of Cd(II) onto HARM (Table 8). The positive *ΔH*^0^ value confirmed the endothermic nature of the process while the positive value of *ΔS*^0^ indicated an increase in the degree of freedom of this adsorbing system, which may be attributed to the cation exchange process and specific sorption process. Because during these processes the amount of Na^+^ and H^+^ released was more than the amount of Cd^2+^ sorbed by red mud^40^, however there was no remarkable change in the structure of HARM during the sorption process because *ΔS*^0^ was not considerable^14^.

11 $$\Delta {G}^{{\rm{0}}}=-\,{RTlnb}$$

12 $$\Delta {{G}}^{0}=\Delta {H}^{0}-T\Delta {{S}}^{0}$$

where *R* is the universal gas constant (8.314 J∙mol^−1^∙K^−1^), T is temperature in Kelvin, the Langmuir constant, *b* is simply recalculated as dimensionless.

**Table 8:** Thermodynamic parameters of Cd(II) sorption onto heat-activated red mud.

Temp. (°C)	*ΔG*^0^ (kJ∙mol^−1^)	*ΔH* ^0^ (kJ∙mol^−1^)	*ΔS*^0^ (kJ∙mol^−1^)
20	−29.20	3.61	0.11
30	−30.22		
40	−31.43		

## Conclusion

In this study, the heat-activated red mud (HARM) sorbent was prepared and characterized by XRD and other physicochemical methods. To characterize the Cd(II) sorption behavior of HARM under various process conditions, a three-factor, three-level Box-Behnken experimental design combined with response surface methodology (RSM) was employed to develop a mathematical model. Based on the RSM result, initial pH was identified as the most important process parameter and had a positive correlation with sorption. Greater Cd(II) sorption exhibited at higher pH could be attributed to weaker H^+^ competition with Cd(II) cations for HARM sorption sites at lower H^+^ concentration. It might also be the result of enhanced Cd(II) sorption via ion exchange due to increases in monovalent Cd species from hydrolysis at higher pH. Moreover, less H^+^ in the solution enhanced specific sorption through decreasing *ΔG* of the specific sorption. Isotherm and thermodynamic studies demonstrated that sorption was a favorable and spontaneous process. A positive *ΔH*^0^ value indicated the endothermic nature of the process, which was consistent with the increase in sorption at higher temperature; however the sorption was independent of reaction temperature at lower initial Cd concentrations (C_0_ < 11 mg∙L^−1^). It may be because when compared with the lower Cd(II) sorbates concentration, sorption sites on the surface of HARM were sufficient, causing the decrease of sorption enhancement by increasing temperature.

## Electronic supplementary material

Supplementary Information

## Data Availability

All data generated or analyzed during this study are included in this published article and its Supplementary Information file.

## References

[1] Wang S, Ang HM, Tadé MO (2008). Novel applications of red mud as coagulant, adsorbent and catalyst for environmentally benign processes. Chemosphere.

[2] Ahmed MJK, Ahmaruzzaman M (2016). A review on potential usage of industrial waste materials for binding heavy metal ions from aqueous solutions. J. Water Process Eng..

[3] Paramguru R, Rath P, Misra V (2004). Trends in red mud utilization – a review, Mineral Process. Extr. Metall. Rev..

[4] Lopez E (1998). Adsorbent properties of red mud and its use for wastewater treatment. Water Res..

[5] Linh VD, Chi TD, Hai HT (2016). The comparison of red mud modification by acid and heat for phosphate removal from aqueous solution. Int. J. Innovative Stud. Sci. Eng. Technol..

[6] Tangde V, Prajapati S, Mandal B, Kulkarni N (2017). Study of kinetics and thermodynamics of removal of phosphate from aqueous solution using activated red mud. Int. J. Environ. Res..

[7] Çengeloğlu Y, Kır E, Ersöz M (2002). Removal of fluoride from aqueous solution by using red mud. Sep. Purif. Technol..

[8] Cengeloglu Y, Tor A, Arslan G, Ersoz M, Gezgin S (2007). Removal of boron from aqueous solution by using neutralized red mud. J. Hazard. Mater..

[9] Luo L (2011). New insights into the sorption mechanism of cadmium on red mud. Environ. Pollut..

[10] Smičiklas I (2014). Effect of acid treatment on red mud properties with implications on Ni(II) sorption and stability. Chem. Eng. J..

[11] Vaclavikova M, Misaelides P, Gallios G, Jakabsky S, Hredzak S (2005). Removal of cadmium, zinc, copper and lead by red mud, an iron oxides containing hydrometallurgical waste. Stud. Surf. Sci. Catal..

[12] Gupta SS, Bhattacharyya KG (2006). Removal of Cd(II) from aqueous solution by kaolinite, montmorillonite and their poly (oxo zirconium) and tetrabutylammonium derivatives. J. Hazard. Mater..

[13] Luo L, Ma YB, Zhang SZ, Wei DP, Zhu YG (2009). An inventory of trace element inputs to agricultural soils in China. J. Environ Manage..

[14] Khan TA, Chaudhry SA, Ali I (2015). Equilibrium uptake, isotherm and kinetic studies of Cd(II) adsorption onto iron oxide activated red mud from aqueous solution. J. Mol. Liq..

[15] Satarug S, Vesey DA, Gobe GC (2017). Health risk assessment of dietary cadmium intake: do current guidelines indicate how much is safe?. Environ. Health Perspect..

[16] Uddin MK (2017). A review on the adsorption of heavy metals by clay minerals, with special focus on the past decade. Chem. Eng. J..

[17] Altundoğan HS, Tümen F (2003). Removal of phosphates from aqueous solutions by using bauxite. II: the activation study. J. Chem. Technol. Biotechnol..

[18] Smiljanić S, Smičiklas I, Perić-Grujić A, Lončar B, Mitrić M (2010). Rinsed and thermally treated red mud sorbents for aqueous Ni^2+^ ions. Chem. Eng. J..

[19] Chen H, Zheng J, Zhang Z, Long Q, Zhang Q (2016). Application of annealed red mud to Mn^2+^ ion adsorption from aqueous solution. Water Sci. Tech..

[20] Milonjić S, Ruvarac AL, Šušić M (1975). The heat of immersion of natural magnetite in aqueous solutions. Thermochim. Acta.

[21] Hanrahan G, Lu K (2006). Application of factorial and response surface methodology in modern experimental design and optimization. Crit. Rev. Anal. Chem..

[22] Yetilmezsoy K, Demirel S, Vanderbei RJ (2009). Response surface modeling of Pb (II) removal from aqueous solution by Pistacia vera L.: Box – Behnken experimental design. J. Hazard. Mater..

[23] Benyounis KY, Olabi AG, Hashmi MSJ (2005). Effect of laser welding parameters on the heat input and weld-bead profile. J. Mater. Process. Technol..

[24] Lin S (2017). Study on the influence of thiolation on the adsorption and magnetic recovery of superparamagnetic nanoadsorbents for Cd^2+^ removal. Appl. Surf. Sci..

[25] Antunes MLP (2011). Red mud from Brazil: thermal behavior and physical properties. Eng. Chem. Res..

[26] Brown, R. B. Soil texture. (ed. Soil and Water Science Department, University of Florida Cooperative Extension Service, Institute of Food and AgricultureSciences, EDIS) (1998).

[27] Tripathi P, Srivastava VC, Kumar A (2009). Optimization of an azo dye batch adsorption parameters using Box – Behnken design. Desalin..

[28] Liu HL, Lan YW, Cheng YC (2004). Optimal production of sulphuric acid by Thiobacillus thiooxidans using response surface methodology. Process Biochem..

[29] Geyikçi F, Kılıç E, Çoruh S, Elevli S (2012). Modelling of lead adsorption from industrial sludge leachate on red mud by using RSM and ANN. Chem. Eng. J..

[30] Lilliefors HW (1967). On the Kolmogorov-Smirnov Test for normality with mean and variance unknown. J. Am. Stat. Assoc..

[31] Srivastava VC, Mall ID, Mishra IM (2006). Equilibrium modelling of single and binary adsorption of cadmium and nickel onto bagasse fly ash. Chem. Eng. J..

[32] Wang C, Liu J, Zhang Z, Wang B, Sun H (2012). Adsorption of Cd(II), Ni(II), and Zn(II) by tourmaline at acidic conditions: kinetics, thermodynamics, and mechanisms. Ind. Eng. Chem. Res..

[33] Lackovic K, Angove MJ, Wells JD, Johnson BB (2003). Modeling the adsorption of Cd (II) onto Muloorina illite and related clay minerals. J. Colloid Interface Sci..

[34] Srivastava P, Singh B, Angove M (2005). Competitive adsorption behavior of heavy metals on kaolinite. J. Colloid Interface Sci..

[35] Serrano S, O’Day PA, Vlassopoulos D, García-González MT, Garrido F (2009). A surface complexation and ion exchange model of Pb and Cd competitive sorption on natural soils. Geochim. Cosmochim. Acta.

[36] Langmuir I (1918). The adsorption of gases on plane surfaces of glass, mica and platinum. J. Am. Chem. Soc..

[37] Weber TW, Chakravorti RK (1974). Pore and solid diffusion models for fixed‐bed adsorbers. AIChE J..

[38] Gupta VK, Sharma S (2002). Removal of cadmium and zinc from aqueous solutions using red mud. Environ. Sci. Technol..

[39] Mishra PC, Patel RK (2009). Removal of lead and zinc ions from water by low cost adsorbents. J. Hazard. Mater..

[40] Crist RH, Martin JR, Crist DR (2002). Heavy metal uptake by lignin: comparison of biotic ligand models with an ion-exchange process. Environ. Sci. Technol..

[41] Mohan S, Gandhimathi R (2009). Removal of heavy metal ions from municipal solid waste leachate using coal fly ash as an adsorbent. J. Hazard. Mater..

[42] He JY (2017). Rapid adsorption of Pb, Cu and Cd from aqueous solutions by β-cyclodextrin polymers. Appl. Surf. Sci..

[43] Guo XY, Zhang SZ, Shan XQ (2008). Adsorption of metal ions on lignin. J. Hazard. Mater..

[44] Jia JZ, Liu CK, Wang L, Liang XY, Chai XY (2018). Double functional polymer brush-grafted cotton fiber for the fast visual detection and efficient adsorption of cadmium ions. Chem. Eng. J..

[45] Babić BM, Milonjić SK, Polovina MJ, Kaludierović BV (1999). Point of zero charge and intrinsic equilibrium constants of activated carbon cloth. Carbon.

[46] Kataria N, Garg VK (2018). Green synthesis of Fe_3_O_4_ nanoparticles loaded sawdust carbon for cadmium (II) removal from water: Regeneration and mechanism. Chemophere.

[47] Visa M, Isac L, Duta A (2012). Fly ash adsorbents for multi-cation wastewater treatment. Appl. Surf. Sci..

[48] Koju NK, Song X, Wang Q, Hu ZH, Colombo C (2018). Cadmium removal from simulated groundwater using alumina nanoparticles: behaviors and mechanisms. Environ. Pollut..

[49] Visa M, Duta A (2013). TiO_2_/fly ash novel substrate for simultaneous removal of heavy metals and surfactants. Chem. Eng. J..

[50] Sahu MK, Mandal S, Yadav LS, Dash SS, Patel RK (2016). Equilibrium and kinetic studies of Cd(II) ion adsorption from aqueous solution by activated red mud. Desalin. Water Treat..

[51] Chen GN, Shah KJ, Shi L, Chiang PC (2017). Removal of Cd(II) and Pb(II) ions from aqueous solutions by synthetic mineral adsorbent: Performance and mechanisms. Appl. Surf. Sci..

[52] Tang N (2018). Efficient removal of Cd^2+^ and Pb^2+^ from aqueous solution with amino- and thiol-functionalized activated carbon: Isotherm and kinetics modeling. Sci. Total Environ..

[53] Xiong T (2018). Insight into highly efficient removal of cadmium and methylene blue by eco-friendly magnesium silicate-hydrothermal carbon composite. Appl. Surf. Sci..

