# Supplementary material for: Characteristics of Cadmium Sorption by Heat-Activated Red Mud in Aqueous Solution
Source: Sci Rep. 2018 Sep 10;8:13558. doi: 10.1038/s41598-018-31967-5 (PMC6131504; doi:10.1038/s41598-018-31967-5)
Supplement: Supplementary file 1 — Supplementary Information [file 41598_2018_31967_MOESM1_ESM.docx]

**Characteristics of Cadmium Sorption by Heat-Activated Red Mud in** **Aqueous Solutions**

Tianxue Yang^1, 2^, Lianxi Sheng^1*^, Yongfeng Wang^2^, Kristen N. Wyckoff^2^, Chunguang He^1^, Qiang He^2, 3*^

^1^ State Environmental Protection Key Laboratory of Wetland Ecology and Vegetation Restoration, School of Environment, Northeast Normal University, Changchun, Jilin, China

^2^ Department of Civil and Environmental Engineering, University of Tennessee, Knoxville, Tennessee, USA

^3^ Institute for a Secure and Sustainable Environment, University of Tennessee, Knoxville, Tennessee, USA

*Corresponding Author: Lianxi Sheng*

E-mail: [shenglx@nenu.edu.cn](mailto:shenglx@nenu.edu.cn)

Telephone number: +86 13804462178

*Corresponding Author: Qiang He*

E-mail: [qianghe@utk.edu](mailto:qianghe@utk.edu)

Fax: +1 865 974 2669

Telephone number: +01 865 9746067

| Run | Cd concentration (mg∙L^−1^) | pH | Time (h) | Observed, *q_e_* (mg∙g^−1^) | Predicted, *q_e_* (mg∙g^−1^) | Residual |
| --- | --- | --- | --- | --- | --- | --- |
| 1 | 102 | 2 | 12.5 | 4.52 | 3.94 | 0.58 |
| 2 | 107 | 6 | 24 | 35.40 | 34.92 | 0.48 |
| 3 | 203 | 6 | 1 | 25.09 | 24.92 | 0.17 |
| 4 | 8 | 6 | 24 | 14.68 | 13.27 | 1.41 |
| 5 | 203 | 4 | 12.5 | 9.98 | 10.12 | −0.14 |
| 6 | 12 | 2 | 24 | 1.44 | 1.84 | −0.40 |
| 7 | 12 | 6 | 24 | 18.06 | 14.29 | 3.77 |
| 8  9 | 74  169 | 6  6 | 24  24 | 29.73  42.95 | 28.52  44.74 | 1.21  −1.79 |

**Supplementary Table S1.** Validation set. *q_e_*: The amount of Cd(II) sorbed.

**Supplementary Figure S1.** Determination of the pH_PZC_ value of heat-activated red mud.

**Supplementary** **Figure S2.** Equilibrium data fitting using linear Langmuir at different reaction temperatures. Error bars are standard deviations.

**Supplementary Figure S3.** △G^0^ vs. temperature plot.
